# K_2_Ti_6_O_13_ Nanoparticle-Loaded Porous rGO Crumples for Supercapacitors

**DOI:** 10.1007/s40820-019-0344-3

**Published:** 2019-12-26

**Authors:** Chongmin Lee, Sun Kyung Kim, Hankwon Chang, Hee Dong Jang

**Affiliations:** 1 Department of Nanomaterials Science and Engineering, University of Science and Technology, Yuseong-gu, Daejeon, 34113 Republic of Korea; 2 Resources Utilization Research Center, Korea Institute of Geoscience and Mineral Resources, Yuseong-gu, Daejeon, 34132 Republic of Korea

**Keywords:** Potassium hexa-titanate, Porous graphene crumples, Supercapacitors

## Abstract

One-dimensional alkali metal titanates containing potassium, sodium, and lithium are of great concern owing to their high ion mobility and high specific surface area. When those titanates are combined with conductive materials such as graphene, carbon nanotube, and carbon nanofiber, they are able to be employed as efficient electrode materials for supercapacitors. Potassium hexa-titanate (K_2_Ti_6_O_13_, KTO), in particular, has shown superior electrochemical properties compared to other alkali metal titanates because of their large lattice parameters induced by the large radius of potassium ions. Here, we present porous rGO crumples (PGC) decorated with KTO nanoparticles (NPs) for application to supercapacitors. The KTO NP/PGC composites were synthesized by aerosol spray pyrolysis and post-heat treatment. KTO NPs less than 10 nm in diameter were loaded onto PGCs ranging from 3 to 5 µm. Enhanced porous structure of the composites was obtained by the activation of rGO by adding an excessive amount of KOH to the composites. The KTO NP/PGC composite electrodes fabricated at the GO/KOH/TiO_2_ ratio of 1:3:0.25 showed the highest performance (275 F g^−1^) in capacitance with different KOH concentrations and cycling stability (83%) after 2000 cycles at a current density of 1 A g^−1^.

**Electronic supplementary material:**

The online version of this article (10.1007/s40820-019-0344-3) contains supplementary material, which is available to authorized users.

## Highlights

- K_2_Ti_6_O_13_ nanoparticle (KTO NP)-loaded porous reduced graphene oxide crumples (PGCs) were fabricated.
- The specific capacitance was improved due to the synergy effect between KTO and PGC.
- The KTO NP/PGC composites can be promising electrode materials for supercapacitors.

**Electronic supplementary material:**

The online version of this article (10.1007/s40820-019-0344-3) contains supplementary material, which is available to authorized users.

## Graphical abstract

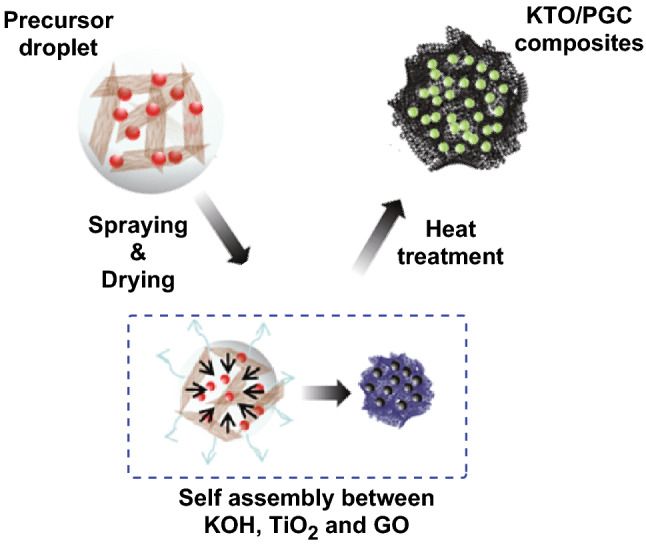

## Introduction

Supercapacitors provide rapid charge/discharge rates, high power density, and good cycle life and are among the most promising energy storage devices [1–4]. However, supercapacitors frequently suffer from low energy density due to their inherently narrow potential window and the low specific capacitance of supercapacitor electrodes [5, 6]. To solve this problem, it is necessary to develop exceptional electrode materials having a large specific surface area, superior electrical conductivity, economical manufacture, and special electrochemical performance. In recent years, much attention has been devoted to one-dimensional tunnel structured alkali metal titanates containing potassium, sodium, and lithium owing to their high ease and speed of their ion mobility, and their high specific surface area [7, 8]. Alkali metal titanates have a monoclinic structure with a chemical formula A_2_Ti_*n*_O_2*n*+1_ (A = Li, K, Na) that is characterized by unique layered (3 < *n* < 5) and tunnel (6 ≤ *n* ≤ 8) crystal structures as well as large specific surface areas [7, 9, 10]. K_2_Ti_6_O_13_ nanoparticles (KTO NPs), in particular, have shown superior electrochemical properties compared to other alkali metal titanates because of their large lattice parameters induced by the large radius of potassium ions [11, 12]. In addition, alkali metal titanates generally need to combine with carbon materials to enhance their electrochemical performances [13]. In previous studies, Zhang et al. prepared K_2_Ti_6_O_13_ nanobeams (K-TNBs)/carbon nanoparticles composites through hydrothermal method for supercapacitor applications. The results showed that the specific capacitance (175 F g^−1^) of the K-TNBs/carbon composites was improved due to the synergy effect between K-TNBs and carbon [14]. Even though enhanced capacitance of KTO NP electrodes through carbon modification, the proposed procedures for improving conductivity are too complicated. Among the various carbon materials, reduced graphene oxide (rGO) is regarded as one of the most promising candidate for supercapacitor by combining with alkali metal titanates because of its excellent electrical conductivity and high surface area [15–19]. In order to enhance the specific capacitance of supercapacitors, detailed studies on KTO NP/rGO composites with exceptional electrochemical properties are required. For example, if porous structure of the composites prepared, the composites provide enlarged surface area of the electrode material for contact with the electrolyte, increase the number of active sites of KTO NP/rGO composites in the electrode material, form an interlacing conductive network, and shorten the diffusion paths. It is then expected to offer potential structural advantages in that electrolyte ions can diffuse to the interior surface efficiently, potentially leading to more effective energy storage devices [20–22]. In this work, porous rGO crumples (PGC) loaded with KTO NP composites were fabricated from a colloidal mixture of TiO_2_, KOH, and graphene oxide (GO) using an aerosol spray pyrolysis and post-heat treatment. While KTO NP/PGC composites were being fabricated, an excessive amount of KOH was employed to obtain enhanced pore structure by activating the rGO. Here, we report the results of our investigation on the effects of the KOH concentration in the KTO NP/PGC composites on their specific surface area, and on measures of the electrical performance of as-prepared composites for supercapacitors.

## Experimental Section

### Synthesis of KTO NP/PCB Composites

An aqueous colloidal suspension of graphene oxide (GO) was prepared from graphite powder (Alfa Aesar, 99.9%) via modified Hummers method [23]. Mixed colloidal solution was stirred at 200 rpm for 1 h in room temperature and used as a precursor including GO with TiO_2_ and KOH. The amount of GO was fixed at 0.5 wt% in colloidal mixture. Therefore, the concentration of precursor was 2.1 wt% for the molar ratio of GO/KOH/TiO_2_ = 1:3:0.25, 1.6 wt% for GO/KOH/TiO_2_ = 1:2:0.25, and 1.1 wt% for GO/KOH/TiO_2_ = 1:1:0.25, respectively. The precursor solution was sprayed by using a standard nozzle (0.7 mm in diameter), and the droplets were then introduced into the preheated chamber (200 °C). The nebulized droplets were rapidly evaporated inside the preheated chamber to produce TiO_2_/KOH/GO crumples powder. The as-fabricated composites were collected in the cyclone. Next, it was further heated at 700, 800, and 900 °C in Ar for 2 h to crystallize the KTO NPs and fabricate porous structure of rGO in the composites. The chemical reaction for the formation of KTO is shown in Eq. 1 [11, 18]:

1 $$6{\text{TiO}}_{2} + 2{\text{KOH}} = {\text{K}}_{2} {\text{Ti}}_{6} {\text{O}}_{13} + {\text{H}}_{2} {\text{O}} .$$

**Fig. 1:**
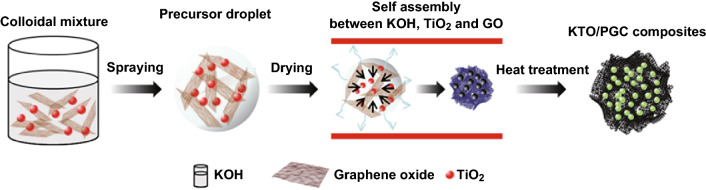
Schematic illustration of the formation of KTO NP/PGC composites from a colloidal mixture of TiO_2_, KOH, and GO via aerosol spray pyrolysis and post-heat treatment

The KTO NP/PGC composite powders were then washed with deionized water to remove residual KOH. Finally, the powders were dried for 2 h at 80 °C. Schematic illustration of fabrication process of KTO NP/PGC composites is shown in Fig. 1.

### Material Characterizations

Transmission electron microscopy (TEM; JEOL, JEM-ARM200F) and field emission scanning electron microscopy (FE-SEM; FEI, Sirion) were used to investigate morphological properties of the electrode materials. The crystalline structures of the prepared materials were analyzed using X-ray diffraction (XRD; Rigaku, RTP 300 RC) with Cu Ka radiation source. Specific surface areas of KTO NP/PGC composites were analyzed by Brunauer–Emmett–Teller method (TriStar 3000, Micromeritics Instrument Corp., Communications Drive, Norcross, GA, USA) using an auto-nitrogen adsorption instrument.

### Electrochemistry Measurements

For preparing the working electrode, the active material (KTO NP/PGC) and polyvinylidene fluoride (PVDF) binder in a weight ratio of 9:1 were mixed with *N*-methyl-2-pyrrolidone (NMP) solvent to obtain slurry. Then the slurry was pasted on carbon paper. Each electrode contained about 4 mg of active material and had a geometric area of about 2.0 cm^2^. Finally, the fabricated electrode was dried at 80 °C for 2 h in a vacuum oven. For full-cell measurements, a two-electrode system of symmetric supercapacitors using 6 M KOH aqueous electrolyte was assembled to test the electrochemical performance and a piece of filter paper (Waterman, GF/C) was used as a separator. Cyclic measurements of cyclic voltammetry (CV), galvanostatic charge–discharge (GCD), and electrochemical impedance spectroscopy (EIS) curves were carried out on an electrochemical workstation (VSP, Bio-logics, USA).

## Results and Discussion

**Fig. 2:**
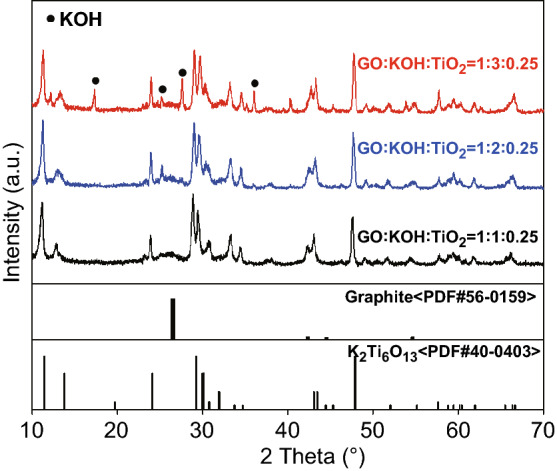
X-ray diffraction patterns of the KTO NP/PGC composites prepared at different weight ratios of GO and KOH (reaction temperature: 200 °C, gas flow rate: 10 L min^−1^, post-heat treatment: 700 °C, 2 h)

**Fig. 3:**
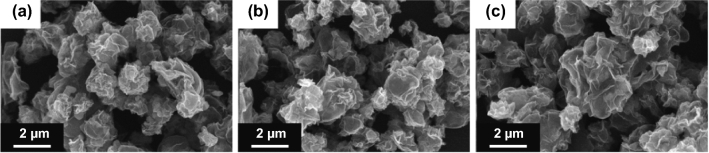
FE-SEM of the KTO NP/PGC composites prepared at different weight ratios (GO:KOH:TiO_2_) of **a** 1:1:0.25, **b** 1:2:0.25, and **c** 1:3:0.25 (reaction temperature: 200 °C, gas flow rate: 10 L min^−1^, post-heat treatment: 700 °C, 2 h)

**Fig. 4:**
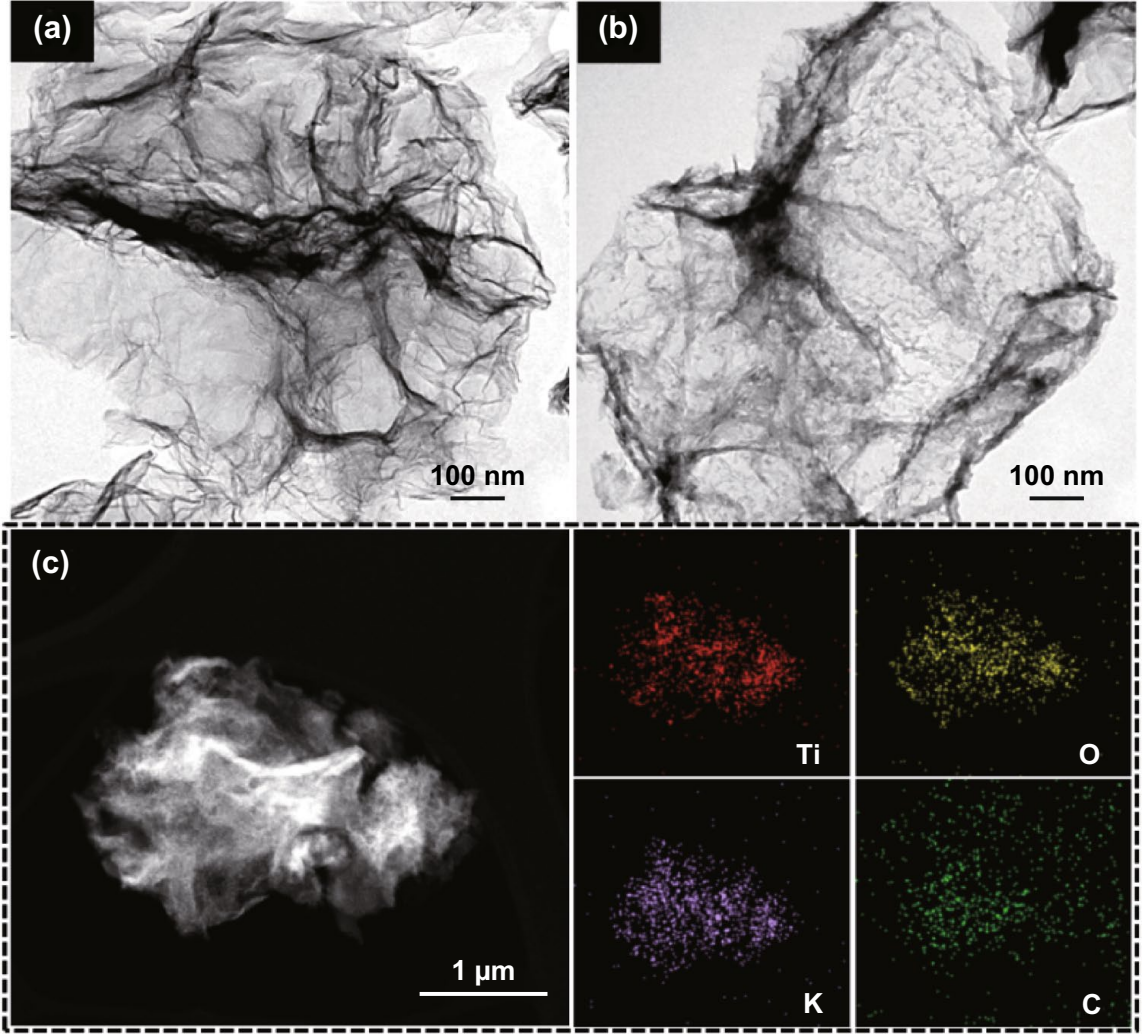
TEM images of the **a** GR crumples, **b** KTO NP/PGC composites prepared at weight ratio of GO:KOH:TiO_2_ for 1:3:0.25 (reaction temperature: 200 °C, gas flow rate: 10 L min^−1^, post-heat treatment: 700 °C, 2 h), **c** EDS elemental mapping analysis of KTO NP/PGC composites

**Fig. 5:**
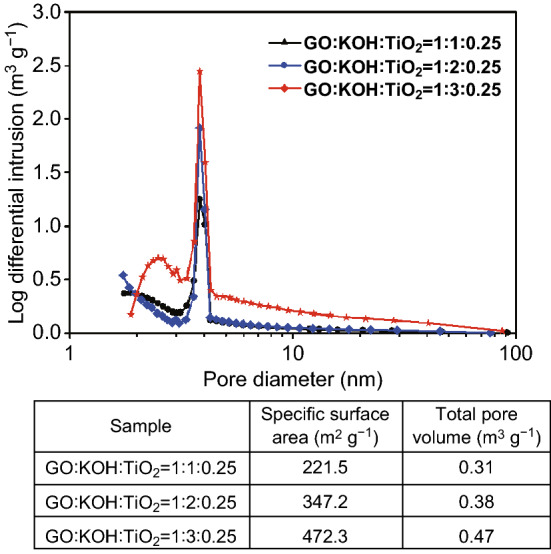
Specific surface area of the KTO NP/PGC composites prepared at different weight ratios of GO and KOH (reaction temperature: 200 °C, gas flow rate: 10 L min^−1^, post-heat treatment: 700 °C, 2 h)

The formation of the KTO NP/PGC composites can be explained as follows. The as-prepared GO colloidal solution was added into a mixture including KOH and TiO_2_. KOH and TiO_2_ were closely integrated on the surface of the GO nanosheets in the KOH/TiO_2_/GO solution. The mixture solution was sprayed by two fluid nozzles and introduced into a chamber preheated to 200 °C. KOH/TiO_2_/GO in droplets was self-assembled to form a crumpled morphology by capillary compression through the evaporation of water in droplets. Figure S1 shows FE-SEM images, XRD, and pore-size distribution of the as-fabricated KOH/TiO_2_/GO composites, while the weight ratio of GO/KOH/TiO_2_ in the mixture solution was 1:3:0.25. During the following post-heat treatment process, the deposited KOH/TiO_2_ precursor was converted to crystalline KTO NPs, as indicated in Eq. 1. At the same time, GO was transformed into rGO by the thermal reduction and was activated to obtain porous structure by loading with excessive KOH (Fig. S2). Figure S3 shows the dependence of the BET specific surface area of KTO NPs/PGC with respect to temperature, for the samples with excessive KOH (relative to GO, i.e., GO/KOH = 1:3). When the temperature was 700 °C, the BET specific surface area reached a maximum of 476 m^2^ g^−1^. Further increase in temperature to 800 °C yielded a specific surface area of 413 m^2^ g^−1^, and at 900 °C, the specific surface area was 238 m^2^ g^−1^. This may be due to the collapse of the porous structure during the high-temperature activation process. It was demonstrated that 700 °C was the optimum temperature for high activity. As shown in Fig. 2, the crystalline structure of the as-synthesized KTO NP/PGC composites was characterized using XRD. The XRD diffraction peaks for the composites corresponded to the K_2_Ti_6_O_13_ phase (JCPDS No. 40-0403) [24]. No diffraction peaks corresponding to rGO, TiO_2_, and KOH were observed in the XRD pattern of the composites in the low KOH concentration. However, small diffraction peaks of KOH were observed in the high KOH concentration because of the residual KOH in the composites. Figure 3 presents the FE-SEM images of the KTO NP/PGC composites with different GO/KOH/TiO_2_ weight ratios of 1:1:0.25, 1:2:0.25, and 1:3:0.25. In Fig. 3, the composites show a 3D crumpled-paper-like morphology ranging from 3 to 5 µm in size. As the amount of KOH increased, the size of the composite particles increased because higher concentration of KOH played a role in greater expansion of PGC during the post-heat treatment. In case of PGC, the existence of PGC of the composite was confirmed by Raman spectra (Fig. S5). For PGC and PGC-based composites, there are two peaks at about 1350 and 1590 cm^−1^ known as D-band and G-band. The D-band is the first-order zone boundary phonon mode associated with a defect in the PGC or PGC edge, and the G-band is a radial C–C stretching mode of *sp*^2^-bonded carbon [25]. The intensity ratio (*I*_D_/*I*_G_) reflects the degree of defect in the PGC. The *I*_D_/*I*_G_ ratio of the KTO NP/PGC composites was higher than that of PGC, which indicated large defect was originated from the activation of rGO by KOH. Figure 4 shows TEM and EDS images of as-fabricated PGC (Fig. 4a) and KTO NP/PGC composites fabricated at GO/KOH/TiO_2_ of 1:3:0.25 (Fig. 4b, c). Figure 4b shows that KTO NPs less than 10 nm were well dispersed on the surface of the PGC, which was expected to promote the high electrochemical performances of the KTO NP/PGC composites. On the other hand, the existence of KOH was hard to distinguish with KTO NPs in the TEM image, even though a KOH peak was present in the XRD analysis in Fig. 2. The residual KOH on the surface of the PGC is considered to have no significant effect on the electrochemical characteristics because KOH was used as the electrolyte in this study. Additionally, the presence of Ti and K in the KTO NP/PGC composites can be further confirmed by EDS analysis as presented in Fig. 4c. The bright spots correspond to the presence of the elements Ti and K, respectively. Figure 4 indicates that Ti and K are distributed uniformly throughout the whole area. It is noted that this crumpled structure is beneficial for diffusing electrolyte ions into the composites and then it provides a promising structure of supercapacitor electrode materials for enhancing electrochemical properties. Figure 5 shows that the pores of KTO NP/PGC composites have two main domains centered at 2–3 nm and 3.5–4.5 nm in high KOH concentration. The specific surface area and pore volume of the composites after post-heat treatment at 700 °C increased with increasing concentration of KOH from 222 to 472 m^2^ g^−1^ and from 0.31 to 0.37 m^3^ g^−1^, respectively. The weight percentage of KTO NPs in the composites (GO/KOH/TiO_2_ = 1:3:0.25) is determined by TGA as shown in Fig. S4. The experiments were performed until 900 °C at a heating rate of 10 °C min^−1^ in air. Under this condition, the PGC was burned up, while KTO NPs were left. Accordingly, the mass ratio of KTO NPs in the KTO NP/PGC composites can be estimated as 46%. It was a little higher than the expected amount of KTO NPs in the precursor because the weight fraction of PGC in the composites decreased due to the formation of pores in the PGC. The composites with high specific surface area are expected to show better rate performance and higher specific capacitance when used as electrode materials for supercapacitors. It is noted that the unique self-assembled 3D network structure offers shortened tunnels for ion and electron transport and promotes the adsorption/desorption of ions. In addition, the high rate performance can be attributed to the increase in the electrode–electrolyte contact area and an acceleration of the ion and electron transport along the surface of rGO [26].

**Fig. 6:**
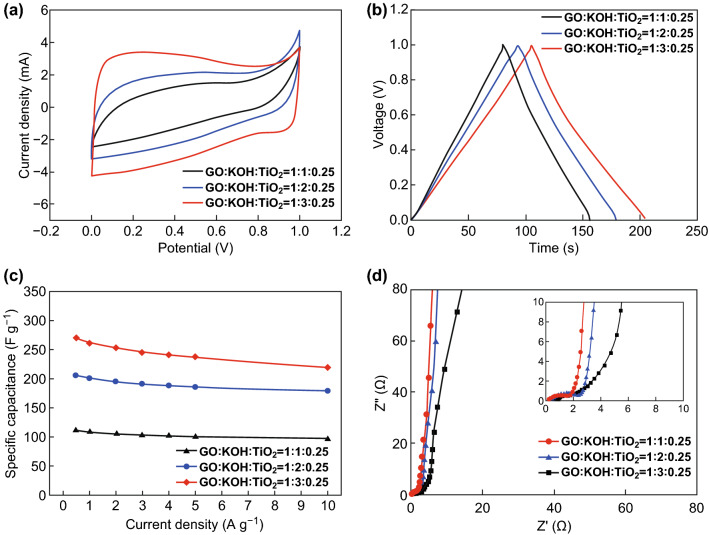
**a** CV curves, **b** GCD curves, **c** specific capacitances, **d** EIS curves of the KTO NP/PGC composites prepared at different weight ratios of GO and KOH, measured using a symmetric two-electrode system

To verify the advantage of the composite electrodes and to explore their promising applications in supercapacitors, CV, GCD, and EIS were measured in a symmetric two-electrode electrochemical system using 6 M KOH aqueous electrolyte from 0 to 1 V. Figure 6a exhibits the CV profiles of a KTO NP/PGC composite electrode measured at a scan rate of 10 mV s^−1^. The CV curves of the composite electrodes were slightly distorted from their ideal rectangular shape, suggesting pseudo-capacitive behavior of the electrode materials. The GCD curves of the composite electrodes in the working potential window of 0–1.0 V are shown in Fig. 6b. GCD measurements were conducted at the current density of 0.5 A g^−1^. When we compare capacitances of composites with different specific surface area, the KTO NP/PGC composites with higher specific surface area exhibited higher specific capacitance (Fig. 6c). The capacitance was 110, 205, and 275 F g^−1^ for GO/KOH/TiO_2_ ratios of 1:1:0.25 (221.5 m^2^ g^−1^), 1:2:0.25 (347.2 m^2^ g^−1^), and 1:3:0.25 (472.3 m^2^ g^−1^), respectively, in an aqueous, symmetric, two-electrode electrochemical system. GCD experiments were performed at the current density of 0.1 A g^−1^. The specific capacitance was also obtained at different current densities to estimate the rate capability of the KTO NP/PGC composite electrodes (Fig. 6c). The capacitance of KTO NP/PGC composites fabricated from GO/KOH/TiO_2_ = 1:3:0.25 was 275, 262, 251, 248, 244, 237, and 223 F g^−1^ at the current density of 0.5, 1, 2, 3, 4, 5, and 10 A g^−1^, respectively. In this case, 86.2% of the specific capacitance was retained as the current density increased from 0.5 to 10 A g^−1^. The composite (GO/KOH/TiO_2_ = 1:3:0.25) exhibited outstanding electrochemical performance due to the large specific surface area and enhanced electron transfer rate, which was originated from the combination of monoclinic structured KTO and highly conductive PGC. It is also considered that the electron moves from a current collect to PGC and flows through the PGC as a highway. Then, the electron finally reaches the electroactive sites of KTO NPs through a miscible interface. The interfacial miscibility in terms of electronic properties enables KTO NP/PGC composites to enhance electrochemical properties [27]. EIS measurements were also conducted to measure the resistance properties of the KTO NP/PGC electrodes. Figure 6d shows a Nyquist plot consisting of a line in the low-frequency region and a semicircle in the high-frequency region. The intercepts of the Nyquist plots on the real axis indicates that the solution resistance is about 0.6 Ω. The interface charge transfer resistance was obtained by the diameter of a semicircle [28]. The interface charge transfer resistance of composites (GO/KOH/TiO_2_ = 1:3:0.25) was smaller than those of other samples. In particular, the KTO NP/PGC composites with higher specific surface area showed lower resistance. This could be attributed to porous structures of the composites and synergy effect by the combination of KTO NPs and PGC, which resulted in low resistance and easy electrolyte diffusion.

**Fig. 7:**
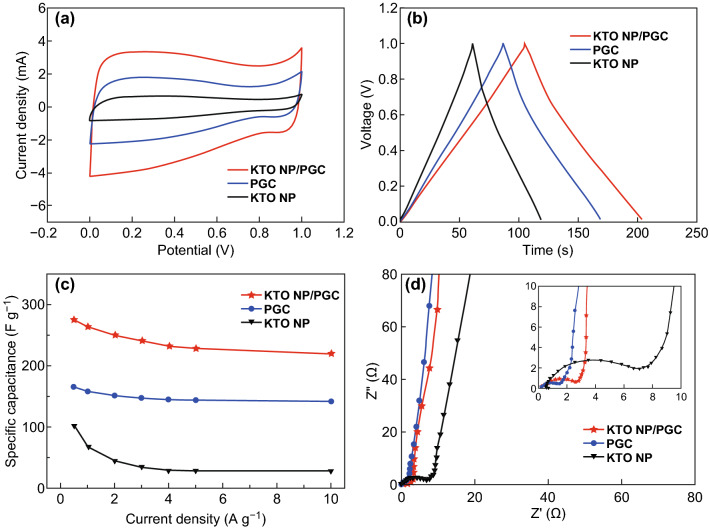
**a** CV curves, **b** GCD curves, **c** specific capacitances, **d** EIS curves of the KTO NPs, PGC, and KTO NP/PGC composites measured using a symmetric two-electrode system

**Fig. 8:**
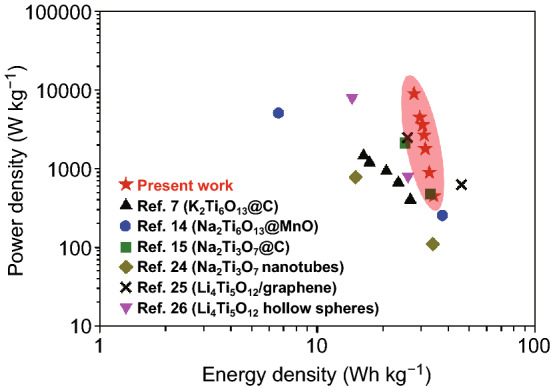
Ragone plot correlating energy density and power density, in comparison with other recently reported supercapacitor devices using alkali metal titanates

The electrochemical performance of the KTO NP/PGC electrode was evaluated at various current densities, and the results are shown in Fig. 7. For comparison, the electrochemical performance of KTO NP and PGC electrodes is also shown in Fig. 7. The KTO NP/PGC composites exhibited the highest specific capacitance of 275 F g^−1^ at a current density of 0.5 A g^−1^, whereas the specific capacitance of KTO NPs and PGC decreased to 102 F g^−1^ and 165 F g^−1^, respectively. Figure S6 shows the long-term cycling stability of 83% at 1 A g^−1^ for 2000 cycles. The cycling stability of the KTO NP/PGC composites is as good as that of the other configurations of symmetric supercapacitors. This further indicated the advantages of the KTO NP/PGC composites as supercapacitor electrode materials. The Ragone plot, presenting energy density and power density, of our work (KTO NP/PGC) and others for symmetric supercapacitor is shown in Fig. 8. The highest energy density of our work was 34.4 Wh kg^−1^ at a power density of 450 W kg^−1^ and the highest power density of 9000 W kg^−1^ at an energy density of 27.9 Wh kg^−1^. Such high output energy and power densities were highly competitive compared to those of the recently reported alkali metal titanate-based supercapacitor devices [7, 14, 15, 29–31].

## Conclusions

We introduced a promising method to fabricate KTO NP/PGC composites having high performance of the electrochemical supercapacitor. The KTO NP/PGC composites with different specific surface area and sizes were synthesized with variation of the KOH concentration. The new composites were composed of KTO NPs less than 10 nm in diameter, which were loaded onto PGC ranging from 3 to 5 µm. The highest specific capacitance of the as-prepared KTO NP/PGC composite electrode was 275 F g^−1^ at the current density of 0.5 A g^−1^ due to enhanced electrical conductivity and specific capacitance by the synergistic effect of PGC and KTO NP. The specific capacitance of activated KTO NP/PGC composites was higher than that of KTO NP/PGC composites that were not activated because the high activity creates more pathways for the electrolyte to penetrate into the bulk material. Considering the achieved electrochemical performance and demonstrated practical application of the KTO NP/PGC-based symmetric supercapacitor device, we strongly believe that KTO NP/PGC composites are a promising electrode material for energy storage in supercapacitor.

## Electronic supplementary material

Below is the link to the electronic supplementary material.

Supplementary material 1 (PDF 397 kb)
