# Supplementary material for: K2Ti6O13 Nanoparticle-Loaded Porous rGO Crumples for Supercapacitors
Source: Nanomicro Lett. 2019 Dec 26;12:10. doi: 10.1007/s40820-019-0344-3 (PMC7770654; doi:10.1007/s40820-019-0344-3)
Supplement: Supplementary file 1 — Supplementary material 1 (PDF 397 kb) [file 40820_2019_344_MOESM1_ESM.pdf]

Supporting information for

$K_2Ti_6O_{13}$ Nanoparticles Loaded porous rGO Crumples for Supercapacitors

Chongmin Lee[1, 2], Sun Kyung Kim[2], Hankwon Chang[1, 2], Hee Dong Jang[1, 2, *]

[1]Department of Nanomaterials Science and Engineering, University of Science and Technology, Yuseong-gu, Daejeon 34113, Republic of Korea

[2]Resources Utilization Research Center, Korea Institute of Geoscience and Mineral Resources, Yuseong-gu, Daejeon 34132, Republic of Korea

*Corresponding author. E-mail: hdjang@kigam.re.kr (Hee Dong Jang)

Supplementary Figures

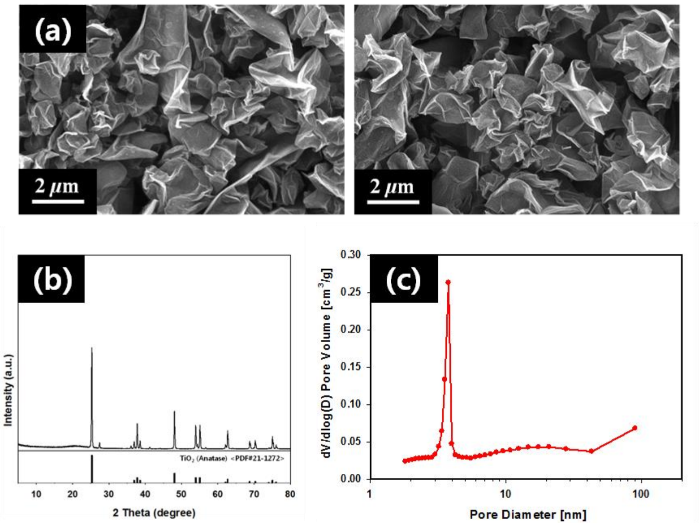

Fig. S1 (**a**) FE-SEM images, (**b**) XRD, and (**c**) pore size distribution of the as- fabricated KOH/TiO_2/GO composites at the weight ratios of GO:KOH:TiO_2 for 1:3:0.25

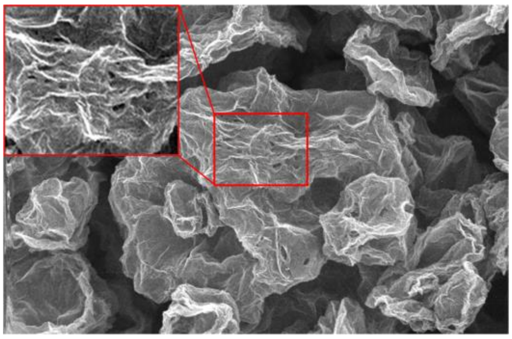

Fig. S2 FE-SEM image of the KTO NP/PGC composites prepared at the weight ratio of GO:KOH:TiO_2 = 1:3:0.25 (reaction temperature: 200°C, gas flow rate: 10 L min^{-1}, post heat treatment: 700°C, 2 h)

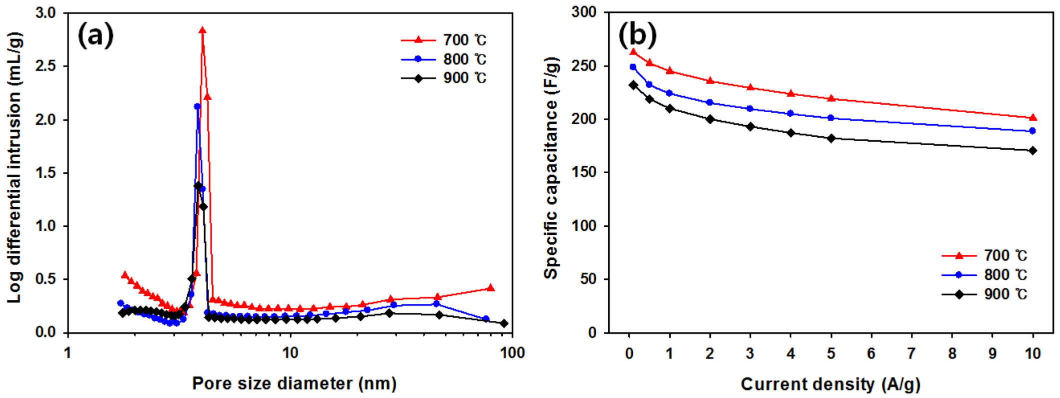

	Specific Surface Area (m^2/g)	Total Pore Volume (m^3/g)	Specific Capacitance (at 0.1 A/g) (F/g)
**700 ℃**	475.5	0.75	263
**800 ℃**	412.7	0.43	248
**900 ℃**	237.7	0.36	232

Fig. S3 Specific surface area and specific capacitance of the KTO NPs/PGC composites (GO:KOH:TiO_2 = 1:3:0.25) prepared at different post heat temperatures (reaction temperature: 200°C, gas flow rate: 10 L min^{-1}, post heat treatment: 600, 700, or 800 °C, 2 h)

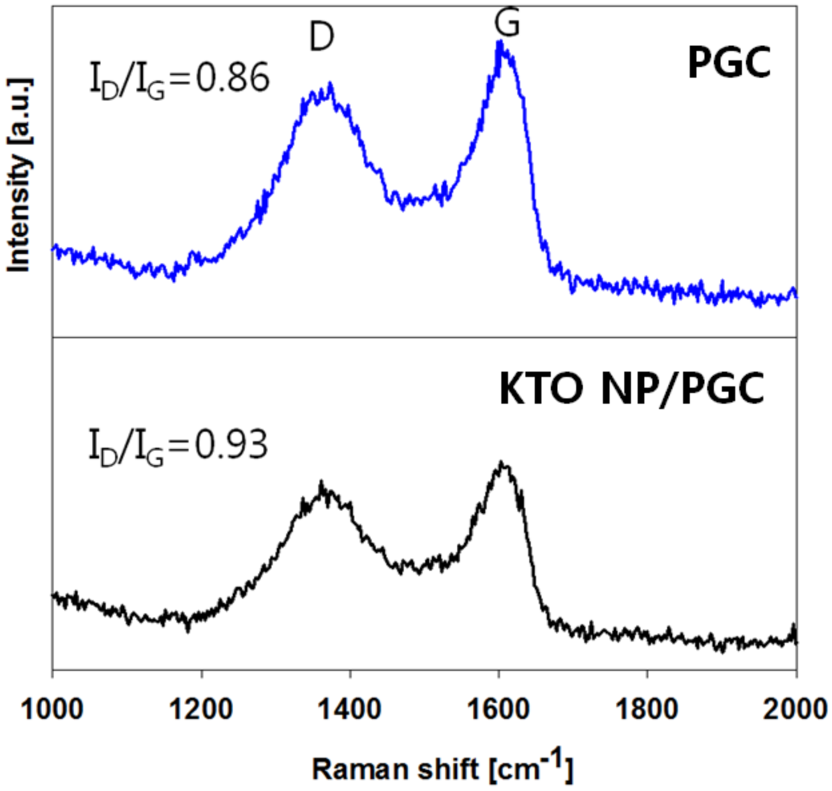

Fig. S4 Raman patterns of the PGC and KTO NP/PGC composites (GO:KOH:TiO_2 = 1:3:0.25)

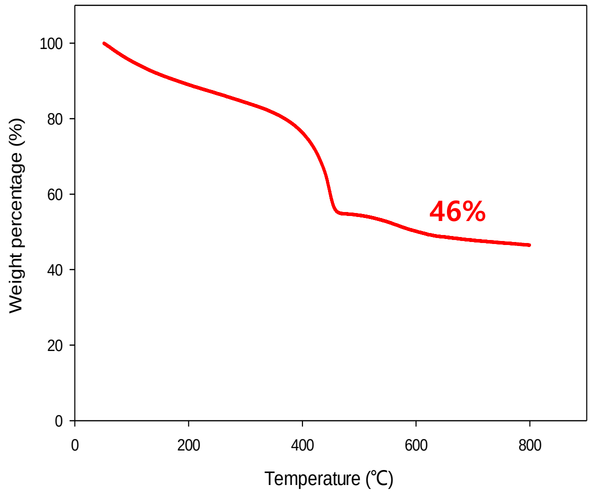

Fig. S5 TG curves of KTO NP/PGC composites (GO:KOH:TiO_2 = 1:3:0.25), under air atmosphere

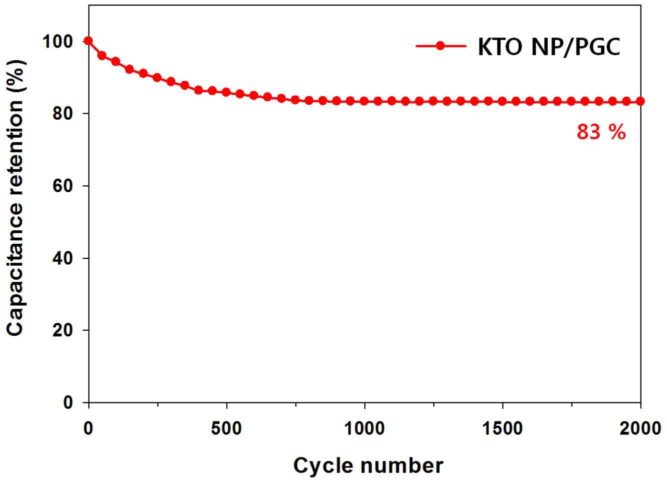

Fig. S6 Cycle stability tested at 1 A g^{-1} for symmetric KTO NP/PGC supercapacitors
